# Faeces‐derived extracellular vesicles participate in the onset of barrier dysfunction leading to liver diseases

**DOI:** 10.1002/jev2.12303

**Published:** 2023-01-28

**Authors:** Lionel Fizanne, Alexandre Villard, Nadia Benabbou, Sylvain Recoquillon, Raffaella Soleti, Erwan Delage, Mireille Wertheimer, Xavier Vidal‐Gómez, Thibauld Oullier, Samuel Chaffron, M. Carmen Martínez, Michel Neunlist, Jérôme Boursier, Ramaroson Andriantsitohaina

**Affiliations:** ^1^ Laboratoire HIFIH UPRES EA 3859 SFR ICAT 4208 Université d'Angers Angers France; ^2^ INSERM UMR1063 Stress Oxydant et Pathologies Métaboliques Faculté de Santé Université d'Angers Université Bretagne Loire Angers France; ^3^ Laboratoire des Sciences du Numérique de Nantes (LS2N) CNRS UMR 6004 – Université de Nantes Nantes France; ^4^ PhyMedExp University of Montpellier INSERM, CNRS Montpellier France; ^5^ Université de Nantes Inserm TENS The Enteric Nervous System in Gut and Brain Diseases IMAD Nantes France; ^6^ Service d'Hépato‐Gastroentérologie et Oncologie Digestive Centre Hospitalier Universitaire d'Angers Angers France

**Keywords:** extracellular vesicles, gut microbiota, intestinal permeability, non‐alcoholic fatty liver disease

## Abstract

The role of extracellular vesicles (EVs) from faeces (fEVs) and small circulating EVs (cEVs) in liver diseases such as non‐alcoholic fatty diseases (NAFLD) and non‐alcoholic steatohepatitis (NASH) has not been demonstrated. fEVs and cEVs of healthy donors, NAFLD and NASH patients were isolated and characterized. The effects of EVs were evaluated in intestinal, endothelial, Kupffer and stellate cells. Non‐muscular myosin light chain kinase (nmMLCK) deficient mice were used in vivo. Bacterial origins of fEVs were analysed by 16s rDNA gene sequencing. fEVs and small cEVs were composed of prokaryotic and eukaryotic origins. Only NASH‐fEVs exerted deleterious effects. NASH‐fEVs increased intestinal permeability and reduced expression of tight junction proteins that were prevented by nmMLCK inhibition, increased endothelial cell permeability and inflammatory cytokines and chemokines requiring TLR4/lipopolysaccharide pathway. NASH‐fEVs and NASH‐cEVs activated profibrotic and proinflammatory proteins of hepatic stellate cells. Treatment with NASH‐fEVs evoked an increase in intestinal permeability in wild type but not in nmMLCK deficient mice. Bacterial origins of fEVs were different between NAFLD and NASH patients and 16 amplicon sequence variants were differentially abundant. We demonstrate that fEVs actively participate in barrier dysfunctions leading to liver injuries underscoring the role of nmMLCK and lipopolysaccharide carried by fEVs.

## 1 INTRODUCTION

Non‐alcoholic fatty liver disease (NAFLD) is currently considered the main chronic liver disease, with a worldwide prevalence of 25% (Younossi et al., 2016). NAFLD is characterized by the presence of steatosis, leading to lipotoxicity (Ibrahim et al., 2011). Liver lipotoxicity precedes liver inflammation and increases the predisposition to the apparition of irreversible non‐alcoholic steatohepatitis (NASH) (George et al., 2018; Lindenmeyer & Mccullough, 2018). NASH is associated with fibrogenesis, resulting in liver impairment and aggravation of the disease. In this regard, NASH could lead to cirrhosis in 21% to 26% of cases (Perumpail et al., 2017) that may evolve into hepatocellular carcinoma in 2.6% and 12.8% of cases (Cholankeril et al., 2017).

The gut microbiota participates in the progression of NAFLD towards NASH and liver fibrosis (Bashiardes et al., 2016; Boursier & Diehl, 2016). Dysbiosis, a shift in bacterial community composition, has been associated with several deleterious effects in NAFLD (Boursier et al., 2016; Brandl & Schnabl, 2017). Dysbiosis was also reported to mediate the destabilization of intestinal and endothelial barriers leading to an increase of intestinal and endothelial permeability (Chen et al., 2015). The intestinal barrier destabilization was associated with a weaker expression of zonula occludens‐1 (ZO‐1). In several diseases with compromised intestinal barriers, the phosphorylation of myosin light chain kinase (MLCK) reduced expression of tight junction (TJ) proteins, and subsequently, increased intestinal permeability (Shen, 2012; Shen et al., 2006). Increased intestinal and endothelial permeabilities trigger greater translocation of pathogen‐associated molecular patterns (PAMPs), like lipopolysaccharide (LPS), in the blood circulation of NAFLD patients (Albillos et al., 2020; Schwenger et al., 2019), that is well corroborated by the positive correlation between endotoxemia and histological NAFLD severity (Pang et al., 2017). Once in circulation, LPS may be transported to the liver, where it activates toll‐like receptor 4 (TLR4). Deletion of TLR4 reduces liver inflammation and fibrogenesis in a TLR4^−/−^ methionine‐choline mouse model (Rivera et al., 2007). Interestingly, PAMPs and other virulence factors can be transported by extracellular vesicles (EVs). EVs are spherical structures produced by eukaryotic and prokaryotic cells and are composed of a cellular or bacterial membrane enclosing cytosolic constituents (Malloci et al., 2019; Toyofuku et al., 2019). Eukaryotic EVs can be divided into three subtypes: apoptotic bodies, large vesicles, and small vesicles. Concerning prokaryotic EVs, gram‐positive and gram‐negative bacteria produce EVs. Bacterial EVs can transfer information to eukaryotic cells, modulate their fate, and elicit deleterious effects in distant host cells (Ellis & Kuehn, 2010; Villard et al., 2021). This is the case for *Pseudomonas aeruginosa* EVs, which elicit a pro‐inflammatory response in macrophages (Bitto et al., 2018) and also for *Campylobacter jejuni* EVs, which cleave the TJ extracellular protein occludin (Elmi et al., 2016). Ultimately, the gut microbiota is a provider of bacterial EVs, which are found in faeces samples. Recently, EVs derived from faeces (fEVs) obtained from high‐fat diet‐fed mice have been reported to promote insulin resistance in myotubes and adipocytes. (Choi et al., 2015) Additionally, mouse fEVs have been demonstrated to induce systemic inflammation in a TLR2/TLR4‐dependent manner in a mouse model (Park et al., 2018). However, no real translational study has been performed on the direct involvement of fEVs in NAFLD/NASH pathophysiology. The aim of this work was to investigate the potential implications of NAFLD/NASH fEVs in the destabilization of the intestinal and endothelial barriers, as well as in liver injuries.

## 2 MATERIALS AND METHODS

### 2.1 Patients

A total of 101 patients with biopsy‐proven NAFLD were included at Angers University Hospital between October 2016 and September 2019. The study protocol conformed to the ethical guidelines of the current Declaration of Helsinki and was approved by the local ethics committee (SNIFF cohort initially approved by CPP OUEST II: 01/19/2010 (CB 2010‐01)) and validated by the National Commission for Computing and Liberties 1998‐001 on 12/20/2017. All patients provided written informed consent before participating in the study.

### 2.2 Animal experiments

All procedures were carried out in accordance with the guidelines and authorization by the French Ministry of Agriculture regulations based on the European Community and were approved by the local ethics committee “Comité d’éthique en expérimentation animale Pays de la Loire”; Apafis#320027‐2019032910558370v5. Please see detailed methods in online Supplemental Data.

## 3 RESULTS

### 3.1 Patients

Patient's characteristics are summarized in Table S1. As expected, NASH patients displayed significantly increased alanine and aspartate aminotransferases compared to nNnN donors.

### 3.2 Characterization of fEVs

**FIGURE 1:**
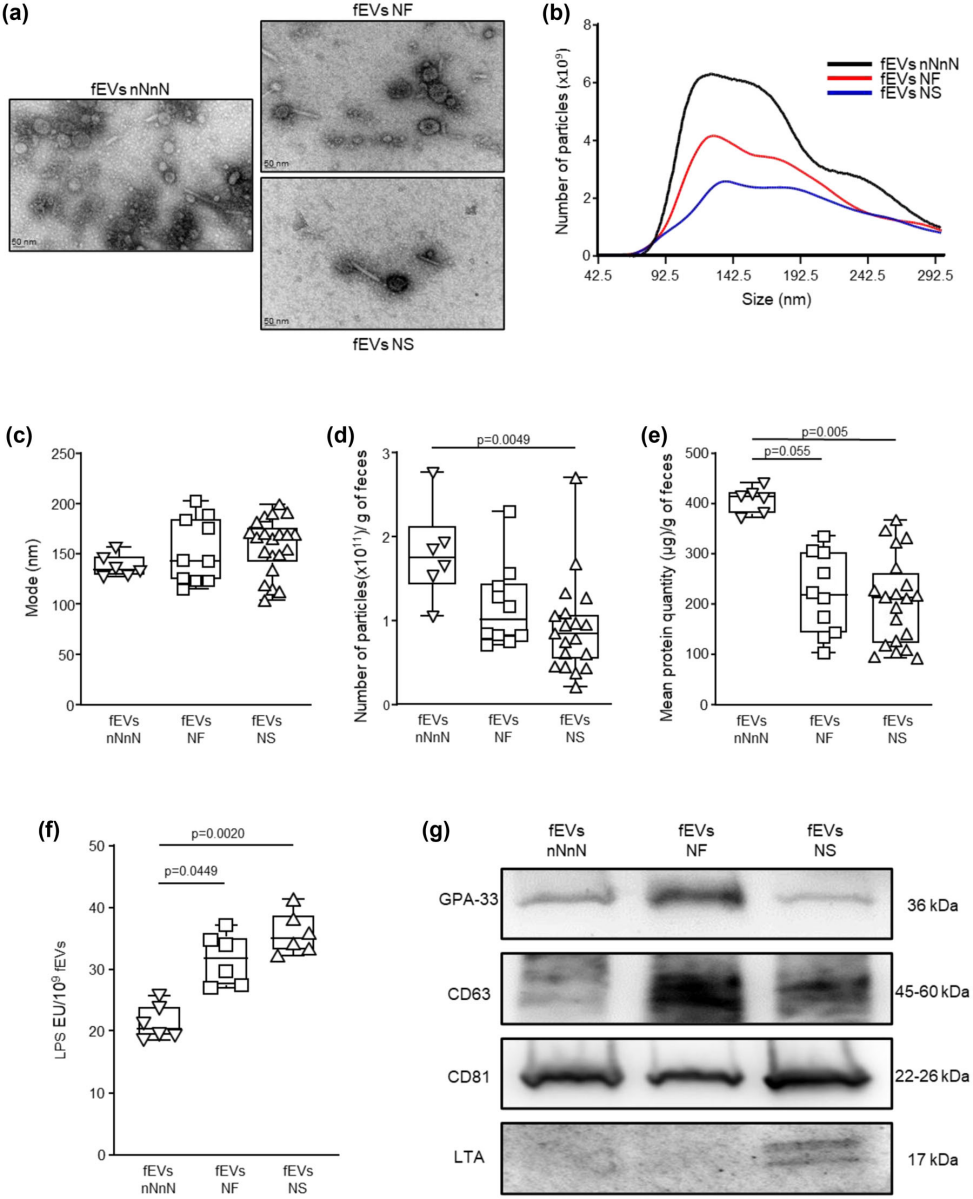
Characterization of faeces‐derived extracellular vesicles (fEVs) from non‐NAFLD/non‐NASH (nNnN) donors or NAFLD (NF)/NASH (NS) patients. (a) Morphology of fEVs in nNnN donor or NF/NS patient samples was assessed by electron microscopy. Scale bar indicates 50 nm. (b) Distribution of size population, (c) mode size and (d) number of particles of fEVs were measured using a nanoparticle tracking analysis. (e) Measurement of protein quantity of EVs from faeces samples. (f) Lipopolysaccharide (LPS) quantification in fEVs samples. (g) Representative western blot showing the presence of cell surface A33 antigen (GPA‐33), CD63, CD81 and lipotechoic acid (LTA) in fEVs samples (*n* = 6–21 patients). Statistical analyses were performed using Kruskal‐Wallis test followed by Dunn's test for multiple comparisons

We confirmed morphology of fEVs in nNnN, NAFLD, and NASH samples using electron microscopy (Figure 1a). nNnN fEVs, NAFLD fEVs, and NASH fEVs displayed similar distribution of size and mode size profiles (Figure 1b and c). nNnN subjects displayed a greater number of particles/gram of faeces compared to NASH patients (Figure 1d). nNnN fEVs had a higher mean protein quantity compared to NAFLD and NASH fEVs (Figure 1e). Interestingly, NAFLD and NASH fEVs had a greater content of LPS compared to nNnN fEVs (Figure 1f). GPA‐33 (a specific marker of enterocytes) and CD63 and CD81 (specific markers of eukaryotic EVs) were present in all types of fEVs, whereas LTA (a specific marker of gram‐positive bacteria) was only expressed in NASH fEVs (Figure 1g).

### 3.3 NASH fEVs enhance epithelial intestinal cell permeability and decrease occludin and ZO‐1 protein expression

**FIGURE 2:**
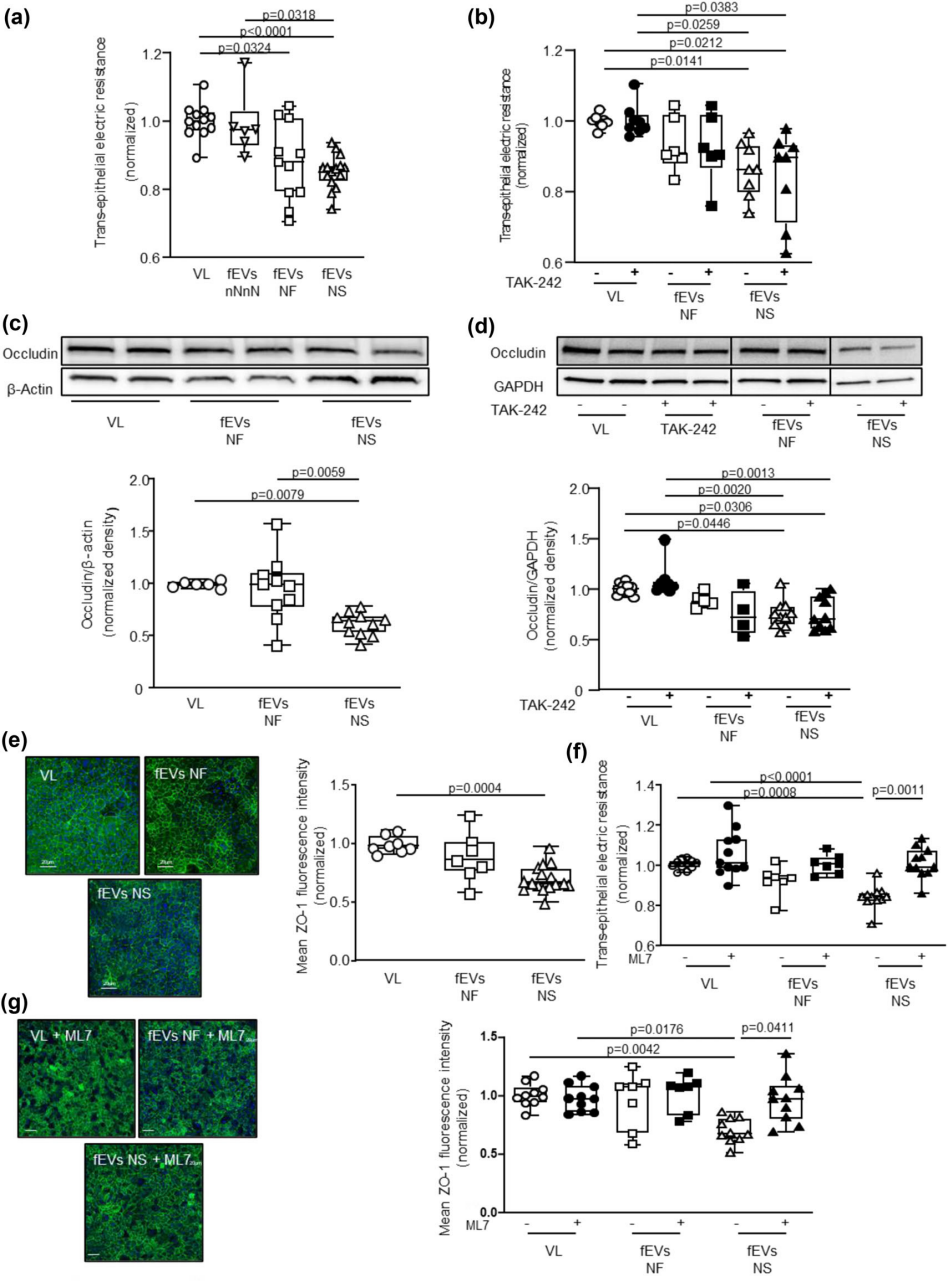
Effects of faeces‐derived extracellular vesicles (fEVs) in intestinal epithelial cell permeability. All experiments were performed in a Caco‐2 cell model stimulated for 24 h by fEVs. The TLR4‐pathway inhibitor, TAK‐242 (3 μM), was added to the cells 30 min prior to fEV stimulation. (a) Epithelial intestinal permeability was measured using trans‐epithelial electric resistance (TEER) before and after fEV stimulation. (b) Permeability was measured using TEER in the absence or in the presence of TAK‐242 and fEVs. (c) Representative western blot and quantification of occludin protein expression following fEV treatment. (d) Representative western blot and quantification of occludin protein expression in the absence or in the presence of TAK‐242 and fEVs. Black line was added on the immunoblots when samples were loaded on the same gel but not side by side. (e) Using immunofluorescence, ZO‐1 (green) protein expression was measured following fEV stimulation. 4′,6‐Diamidino‐2‐phenylindole (DAPI, blue) was used to stain the cell nuclei. Scale bar indicates 20 μm. (f) Epithelial intestinal permeability was measured by trans‐epithelial electric resistance (TEER) in the absence or in the presence of ML‐7 and fEVs. The nmMLCK inhibitor, ML‐7 (10 μM), was added to the cells 30 min prior to fEV stimulation. (g) Immunofluorescence analysis of ZO‐1 (green) in the absence/presence of ML‐7 and fEVs. DAPI (blue) was used to stain the cell nuclei. Scale bar indicates 20 μm (*n* = 6–15). Statistical analysis was performed using the Kruskal‐Wallis test, followed by Dunn's test for multiple comparisons. nNnN, non‐NAFLD/non‐NASH; NF, NAFLD; NS, NASH

NAFLD fEV stimulation decreased the trans‐epithelial electric resistance (TEER) of Caco‐2 cells compared to the vehicle condition. NASH fEV stimulation decreased the TEER values compared to both, the vehicle and nNnN fEV conditions (Figure 2a). The TLR4 inhibitor, TAK‐242 did not modify the effect of NAFLD or NASH fEVs (Figure 2b). NASH fEV, but not NAFLD fEV, stimulation decreased occludin expression in Caco‐2 cells (Figure 2c). TAK‐242 alone, or in the presence of either NAFLD or NASH fEVs, did not modify occludin expression (Figure 2d). To confirm the alteration of TJs proteins, ZO‐1 expression was assessed. ZO‐1 expression was also decreased following NASH fEV stimulation (Figure 2e). These results demonstrate that NASH fEVs increase the Caco‐2 cell permeability, associated with a decrease in occludin and ZO‐1 protein expression, via a TLR4‐insensitive inhibitor pathway.

### 3.4 NASH fEVs enhance the intestinal permeability via a non‐muscular myosin light chain kinase (nmMLCK)‐dependent pathway

The pharmacological inhibitor of nmMLCK, ML‐7, had no effect on TEER, both in control conditions and following NAFLD fEV stimulation (Figure 2f). Interestingly, ML‐7 prevented the decrease in both TEER values and ZO‐1 expression induced by NASH fEVs (Figure 2f and g). Thus, NASH fEVs increased the Caco‐2 cell permeability via an nmMLCK‐dependent pathway.

### 3.5 NASH fEVs increase endothelial permeability and induce endothelial inflammation

**FIGURE 3:**
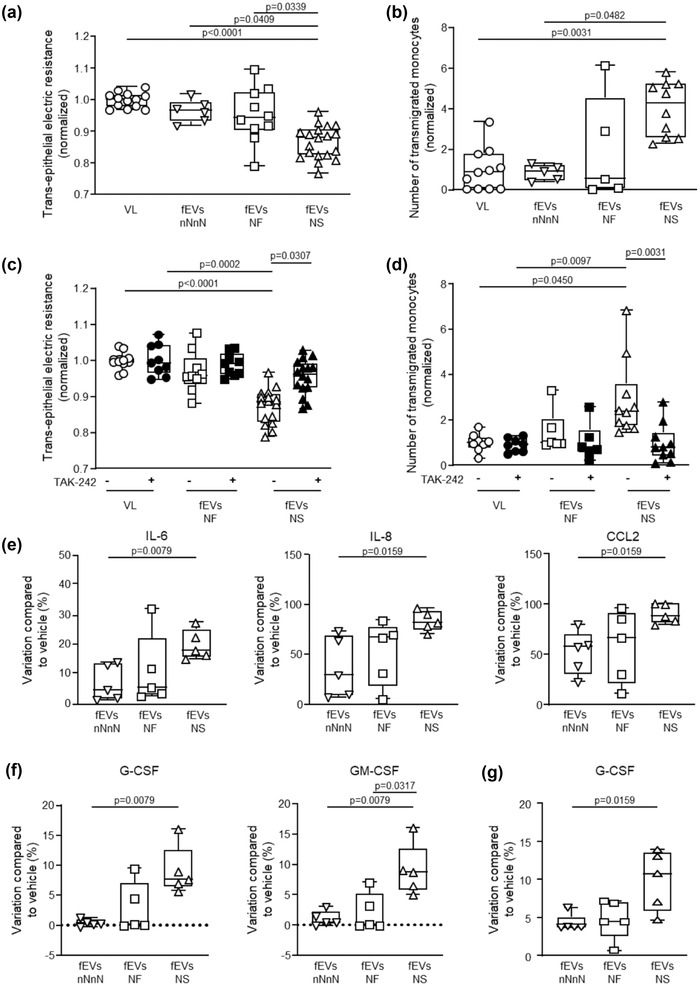
NASH (NS) faeces‐derived extracellular vesicles (fEVs) increase endothelial permeability in a TLR4‐dependent pathway and induce in vitro endothelial inflammation. All experiments were performed in human aortic endothelial cells stimulated for 24 h by fEVs. TLR4‐pathway inhibitor, TAK‐242 (1 μM), was added to the cells 30 min prior to fEV stimulation. (a) Endothelial permeability was measured using trans‐epithelial electric resistance (TEER) before and after fEV stimulation. (b) Transmigration of THP‐1 monocytes through an endothelial monolayer induced by fEVs. Transmigrated monocytes were counted by flow cytometry. (c) Endothelial permeability was measured using TEER in the absence or in the presence of TAK‐242 and fEVs. (d) Transmigration of THP‐1 monocytes through an endothelial monolayer induced in absence/presence of TAK‐242 and fEVs. (e–g) As increased endothelial permeability is often associated with endothelial inflammation, cytokine production was assessed using a cytokine array. Only differentially expressed proteins are represented. (e) Measurement of the variation of protein expression of pro‐inflammatory cytokines IL‐6 and IL‐8, chemokine (C‐C motif) ligand 2 (CCL2), (f) granulocyte colony‐stimulating factor (G‐CSF) and granulocyte‐macrophage colony‐stimulating factor (GM‐CSF). (g) Production of cytokines was also assessed using a cytokine array in a human hepatic sinusoidal endothelial cell model. Measurement of the variation in G‐CSF protein expression (*n* = 5–15). Statistical analysis was performed using the Kruskal‐Wallis test, followed by Dunn's test for multiple comparisons. nNnN, non‐NAFLD/non‐NASH; NF, NAFLD; NS, NASH

In HAoECs, nNnN and NAFLD fEVs had no effect on the TEER values or monocyte transmigration; however, NASH fEVs decreased TEER values compared to vehicle, nNnN, and NAFLD fEVs, and increased monocyte transmigration compared to vehicle and nNnN fEV conditions (Figure 3a,b). TAK‐242 did not modify endothelial permeability and TEER values or monocyte transmigration following NAFLD fEV stimulation. Interestingly, TAK‐242 prevented the decrease in TEER values (Figure 3c) and counteracted the increase in monocyte transmigration (Figure 3d) induced by NASH fEVs. NAFLD fEV stimulation did not modify chemokine/cytokine production compared to nNnN fEV stimulation (Figure 3e–g). In contrast, NASH fEV stimulation induced a significant increased production of the pro‐inflammatory cytokines IL‐6 and IL‐8 (Figure 3e), chemokine (C‐C motif) ligand 2 (CCL2) (Figure 3e), granulocyte‐colony stimulating factor (G‐CSF), and granulocyte‐macrophage colony‐stimulating factor (GM‐CSF) (Figure 3f) compared to stimulation with nNnN fEVs. An increased production of IL‐6 and CCL2 following NASH fEV stimulation was confirmed using a multiplex assay. In addition, cytokine array analyses revealed that pharmacological inhibition of TLR4 receptor with TAK‐242, but not nmMLCK with ML‐7, tended to decrease the ability of NASH fEVs to increase IL‐6, IL‐8, CCL2 and CXCL1 (Figure S1). Thus, it is likely that NASH fEVs induced endothelial permeability, monocyte transcytosis and inflammation by acting on TLR4, probably via the LPS that they carried.

In HHSECs, NAFLD and NASH fEVs did not significantly modify IL‐6, IL‐8, and CCL2 expression compared to nNnN fEV stimulation (data not shown). However, NASH fEVs increased G‐CSF production compared to nNnN fEVs (Figure 3f).

These results suggest that NASH fEVs increase the endothelial permeability via a mechanism sensitive to TLR4 inhibition and endothelial inflammation.

### 3.6 NASH fEVs increase the production of extracellular matrix proteins and chemokines/cytokines production in hepatic stellate (LX‐2 cells), alter hepatocyte integrity and decrease ZO‐1 and occludin protein levels

**FIGURE 4:**
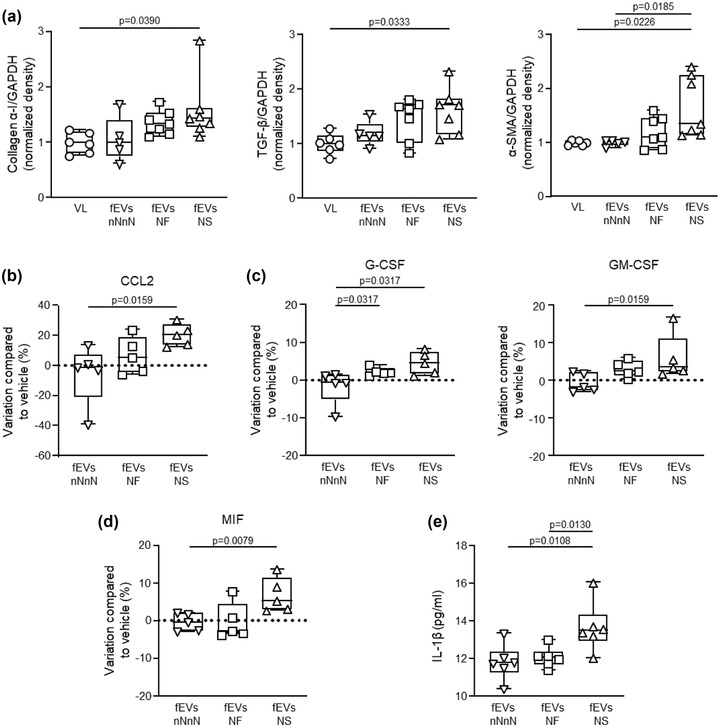
NASH (NS) faeces‐derived extracellular vesicles (fEVs) activate in vitro stellate cells and increase cytokines involved in fibrogenesis. All experiments were performed in a LX‐2 cell model stimulated by fEVs for 24 h. (a) Quantification of the western blot of collagen α‐I, transforming growth factor‐β (TGF‐β), and α‐smooth muscle actin (α‐SMA) protein expression following fEV stimulation. (b–d) Cytokine production was assessed using a cytokine array. Only differentially expressed proteins are represented (b) Measurement of the variation of protein expression of chemokine (C‐C motif) ligand 2 (CCL2), (c) granulocyte colony‐stimulating factor (G‐CSF) and granulocyte‐macrophage colony‐stimulating factor (GM‐CSF) and (d) macrophage migration inhibitory factor (MIF). (e) Quantity of IL‐1β measured using the multiplex assay (*n* = 4–7). Statistical analysis was performed using the Kruskal‐Wallis test followed by Dunn's test for multiple comparisons. nNnN, non‐NAFLD/non‐NASH; NF, NAFLD; NS, NASH

nNnN and NAFLD fEVs did not modify the protein expression of α‐1 type 1 collagen, TGF‐β, or α‐SMA compared to the vehicle. Interestingly, NASH fEV stimulation increased α‐1 type 1 collagen and TGF‐β protein expression compared to the vehicle conditions and increased α‐SMA protein expression compared to the vehicle and nNnN fEVs (Figure 4a).

The activation of LX‐2 cells following NASH fEV stimulation was confirmed by measuring the cytokine/chemokine production. NAFLD fEVs increased the G‐CSF production compared to nNnN fEVs (Figure 4c). NASH fEVs increased the levels of CCL2 (Figure 4b), G‐CSF and GM‐CSF (Figure 4c), and macrophage migration inhibitory factor (MIF) (Figure 4d) compared to nNnN fEVs. Moreover, NASH fEVs increased IL‐1β production compared to both, nNnN and NAFLD fEVs (Figure 4e). Thus, NASH fEVs activate stellate cells and participate in fibrogenesis.

Moreover, NASH fEVs altered hepatocyte integrity, as shown by the decrease in number of bile canaliculi, using 5‐Chloromethylfluorescein diacetate (CMFDA) fluorescence analysis. This effect was associated with a decrease in ZO‐1 and occludin protein levels (Figure S2).

### 3.7 Metabarcoding reveals a differential composition between NAFLD and NASH fEV samples

**FIGURE 5:**
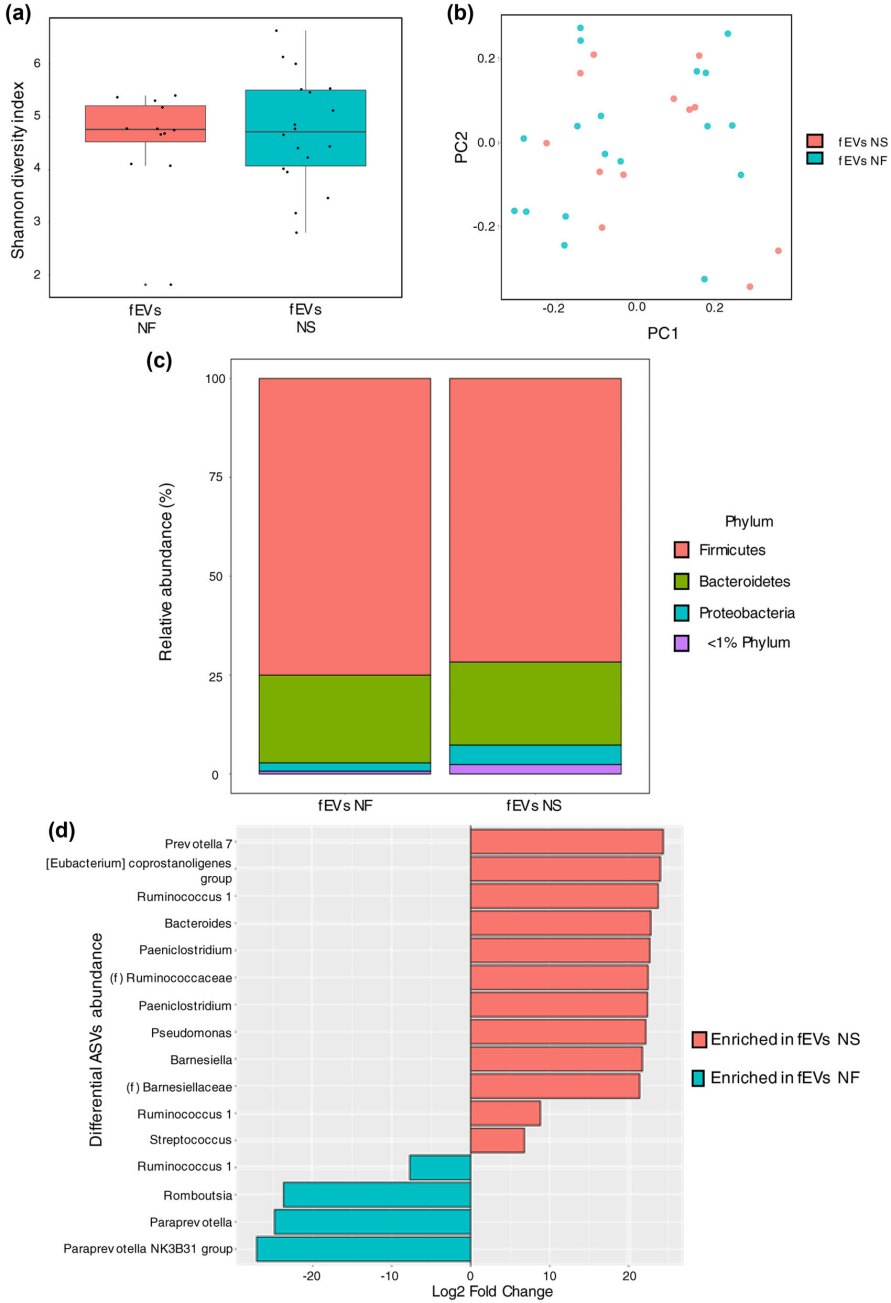
Metagenomic analyses of faeces‐derived extracellular vesicles (fEVs) samples. 16S rDNA was isolated from each fEV sample (NAFLD (NF) and NASH (NS) patients) and sequenced. (a) The alpha diversity between NF and NS fEV samples was measured using the Shannon diversity index. (b) Principal Coordinates Analysis (PCoA) of dissimilarity measured by Bray‐Curtis. (c) Relative abundance in fEVs at the phylum level. (d) Differentially abundant ASVs in NF versus NS samples. Amplicon sequence variants (ASVs) are here either identified by their genus or, when unassigned, their family. Statistical analysis was performed using the Mann‐Whitney test

The bacterial origins of fEVs isolated from NAFLD and NASH patient faeces samples were analysed using 16S rDNA gene sequencing. No significant differences were observed between conditions in terms of α‐diversity (Richness, Figure S3A; or Shannon index, Figure 5a) or β‐diversity (measured using Bray‐Curtis dissimilarity) (Figure 5b). At the phylum level, fEV bacterial compositions were dominated by Firmicutes, Bacteroidetes, and, to a lower extent, Proteobacteria (Figure 5c and Figure S3B). The differential abundance analysis revealed that 12 amplicon sequence variants (ASVs) were enriched in NASH‐associated samples, and 4 were enriched in NAFLD‐associated samples (Figure 5d). The exhaustive list of differentially abundant ASVs is provided in Table S2.

Following this first set of metabarcoding analyses, the bacterial composition of fEV samples was stratified according to NAFLD/NASH presence, which was assessed using NAFLD activity score and the fibrosis severity degree. No significant differences were observed between conditions in terms of α‐diversity (Figures S3d and S4a) or β‐diversity (measured using Bray‐Curtis dissimilarity) (Figure S3c). At the phylum level, no differences were detected between the three groups of patients (Figure S4b). At the genus level, 17 ASVs were enriched in fEVs from patients with a fibrosis grade ≥ 2 compared to fEVs from NASH patients with a fibrosis grade = 0/1 (Figure S4c), and there was a noticeable greater abundance of *Ruminococcus 2* (Figure S4d).

### 3.8 Circulating small EVs (cEVs) isolated from patients with NAFLD and NASH contained prokaryotic EVs and induced cytokine production in stellate cells

**FIGURE 6:**
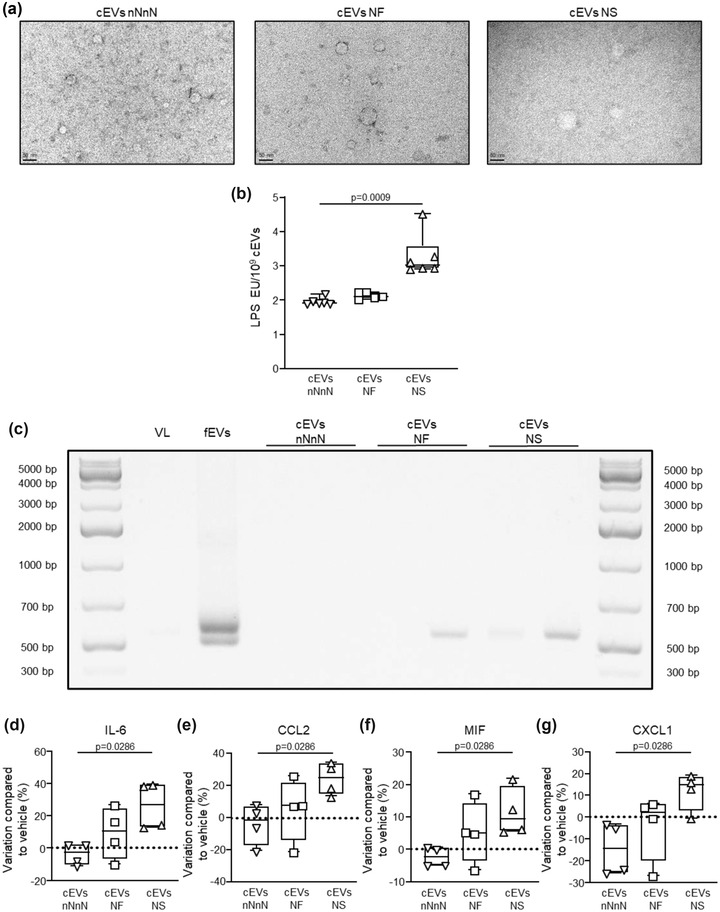
Circulating small extracellular vesicles (cEVs) isolated from non‐NAFLD/non‐NASH (nNnN) donors or NAFLD (NF)/NASH (NS) patients. (a) Morphology of cEVs from nNnN donor or NAFLD/NASH patient samples was assessed by electron microscopy. Scale bar indicates 50 nm. (b) Quantification of LPS carried by cEVs. (c) Presence of 16S rDNA in cEVs was assessed by PCR. (d–g) Production of cytokines by LX‐2 cells after 24 h stimulation with cEVs at circulating concentration assessed using a cytokine array. Only differentially expressed proteins are represented. (d) Measurement of the variation of protein expression of IL‐6, (e) chemokine (C‐C motif) ligand 2 (CCL2), (f) macrophage migration inhibitory factor (MIF) and (g) chemokine (C‐X‐C motif) ligand 1 (CXCL1) (*n* = 4–6) Statistical analysis was performed using the Kruskal‐Wallis test followed by Dunn's test for multiple comparison

cEVs from nNnN donors and NAFLD/NASH patients displayed similar sizes (Figure 6a). The presence of eukaryotic markers TSG‐101, GPA‐33, and CD81 was observed in cEVs using western blot analyses (Figure S5). The LPS concentration was higher in cEVs (Figure 6b) from NASH patients, compared to fEVs from nNnN donors and NAFLD patients. The presence of 16S rDNA was observed in the cEVs from patients with NAFLD and NASH (Figure 6c). In stellate cells, neither nNnN nor NAFLD cEV stimulation modified cytokine/chemokine production (Figure 6d–g). Interestingly, NASH cEV stimulation provoked a greater production of IL‐6 (Figure 6d), CCL2 (Figure 6e), MIF (Figure 6f), and CXCL1 (Figure 6g) compared to nNnN cEV stimulation. Thus, prokaryotic EVs were found in blood circulation and cEVs from NASH patients induce stellate cell inflammation in a similar manner to that observed with fEVs from NASH patients. Finally, NASH cEV stimulation did not significantly modify the IL‐6, IL‐8, MCP1, MIF and/or Serpin1 production in Kupffer cells (Figure S6).

### 3.9 nmMLCK deletion protects against the increase of intestinal permeability induced by NASH fEVs

**FIGURE 7:**
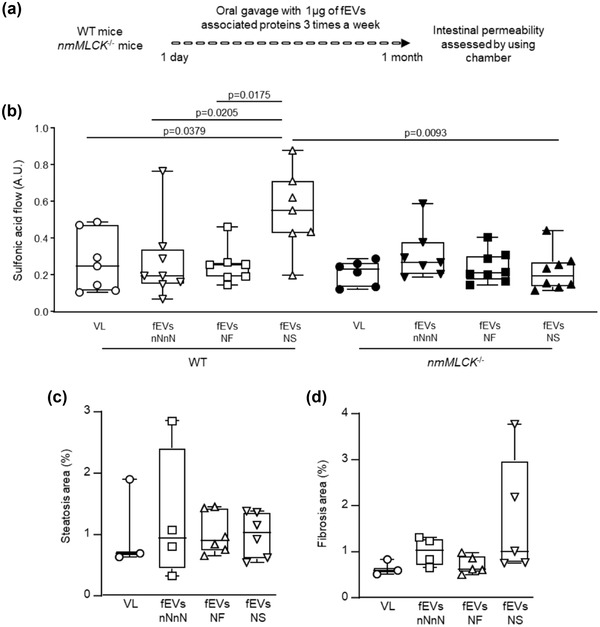
Non muscular myosin light chain kinase (nmMLCK) deletion protects against the increase of intestinal permeability induced by NASH fEVs in vivo. (a) Wild type (WT) or *nmMLCK* ^−/−^ mice (*n* = 6–8) were orally force‐fed 1 μg of fEV‐associated proteins three times a week for 4 weeks. Mice were then sacrificed, and their intestines were collected before measuring the intestinal permeability using an Ussing chamber. (b) The permeability of the ileum of WT and *nmMLCK* ^−/−^ mice was assessed by measuring the sulfonic acid flow through the tissue section. (c, d) Histological assessment of liver steatosis performed after staining of tissue sections with hematoxylin‐eosin and red picrosirius staining. Histological grading of fibrosis was based on the Metavir grading system. Then, areas of steatosis (c) and fibrosis (d) were measured. Statistical analysis was performed using the Kruskal‐Wallis test, followed by Dunn's test for multiple comparisons. VL, vehicle; nNnN, non‐NAFLD/non‐NASH; NF, NAFLD; NS, NASH

In vivo mouse intestinal permeability was measured by using an Ussing chamber, following intragastric administration of fEVs (Figure 7a). Wild‐type mice treated with nNnN and NAFLD fEVs did not display greater sulfonic acid flow in the ileum compared to the vehicle. Wild‐type mice treated with NASH fEVs had a greater sulfonic acid flow in the ileum compared to vehicle, nNnN fEVs, and NAFLD fEVs that was associated with a trend increase in fibrosis but not in steatosis (Figure 7b–d). Importantly, deletion of *nmMLCK* completely protected mice against the increased ileum permeability evoked by NASH fEV treatment (Figure 7b). nNnN, NAFLD, and NASH fEV treatment failed to increase the sulfonic acid flow in the jejunum, proximal colon, and distal colon in both, wild type and nmMLCK^−/−^ mice (Figure S7a–c).

## 4 DISCUSSION

Here, we provide direct evidence that EVs obtained from faeces have prokaryotic and eukaryotic origins, including an intestinal epithelial cell origin. Interestingly, EVs originating from microbiota carrying LPS can be observed in blood. We underscore the severity of fEVs from NASH patients in inducing intestinal barrier dysfunction associated with a decreased TJ protein expression. nmMLCK was the main target involved in the increased intestinal permeability in vitro and in vivo. Moreover, fEVs increased endothelial cell permeability, transmigration of monocytes, and the production of both, proinflammatory cytokines and chemokines from both aortic and hepatic sinusoidal endothelial cells, probably via LPS, which acted on TLR4 receptor. Notably, fEVs and cEVs activated profibrotic and proinflammatory protein production in hepatic stellate cells. Of importance is the fact that fEV bacterial origins are congruent with the gut microbiota composition described in NAFLD and NASH patients. All of these effects highlight the important role played by fEVs in several NASH features observed in these patients.

Complete characterization of fEVs from nNnN, NAFLD and NASH patients revealed that these are composed of EVs originating from prokaryotic, gram‐negative and gram‐positive bacteria, and eukaryotic cells. Regarding gram‐negative bacteria, NASH fEVs carried a greater quantity of LPS. Concerning the eukaryotic origins of fEVs, we demonstrated the presence of small vesicle‐associated proteins, CD81 and CD63 tetraspanins, and of a specific enterocyte protein GPA‐33, a protein implicated in enterocyte proliferation and migration (Bui et al., 2018). Interestingly, we found that cEVs from NASH patients, in addition to eukaryotic markers, carried not only 16S rDNA, but also LPS, demonstrating that EVs from the microbiota can cross the intestinal barrier and reach the bloodstream and, therefore, participate in the pathophysiology of liver diseases.

Present study revealed several differences between fEVs derived from nNnN donors or NAFLD patients, and those derived from NASH patients. We report a lower number of EVs, a lower mean protein quantity, and a higher concentration of LPS in NASH fEVs compared to nNnN fEVs. Our metagenomic analyses highlight the differential abundance of several ASVs between NAFLD fEVs and NASH fEVs. These differences may be the key to fully understand differential functional effects on target cells of the gut‐liver axis. It is important to note that only fEVs from NASH, but not NAFLD patients, exerted significant deleterious effects in the present study.

fEVs may reach the bloodstream through an altered intestinal barrier and move to the liver. This is corroborated by the presence of 16S rDNA in NAFLD and NASH, but not in nNnN cEVs. In addition, we observed an enrichment of LPS in both fEVs and cEVs from NASH patients compared to nNnN donors, suggesting that both fEVs and cEVs can account, at least in part, for the endotoxemia described in patients with liver diseases. Since the increase of permeability takes place only at the ileum, but not jejunum nor colon in mice after NASH fEVs gavage, it is likely that fEVs from NASH patients act mainly at the ileum and therefore cEVs containing 16S rDNA and LPS come from the gut. In accordance with our data, a higher circulating level of LPS and an association between endotoxemia markers and NAFLD severity have been reported in NAFLD/NASH patients (Harte et al., 2010; Pang et al., 2017). Indeed, Tulkens and colleagues reported a lower presence of bacterial EVs in the blood of healthy donors compared to that of patients with a compromised intestinal barrier (Tulkens et al., 2018). In the present study, NASH fEVs disrupted the intestinal barrier by decreasing the expression of TJ proteins, occludin and ZO‐1. Thus, NASH fEVs may destabilize the intestinal barrier by carrying virulence factors absent in nNnN/NAFLD fEVs samples, whose identity remains to be determined.

We previously reported that nmMLCK participates in intermittent hypoxia‐induced endothelial dysfunction, resulting from IL‐6 secretion and endothelial permeability, in addition to its interaction with the NF‐κB pathway in LPS‐induced vascular inflammation (Recoquillon et al., 2015, 2017). Moreover, MLCK alters ZO‐1 distribution and increases occludin endocytosis in Caco‐2 TJs (Jin & Blikslager, 2016; Shen et al., 2006). Additionally, a partial contribution of MLCK to intestinal barrier dysfunction and liver disease has been described after chronic alcohol exposure in mice (Chen et al., 2015). Here, nmMLCK inhibitor prevented the increased intestinal permeability and restored ZO‐1 expression, following NASH fEV stimulation in vitro. Strikingly, *nmMLCK*‐deficient mice were protected against the increased ileal permeability provoked by NASH fEVs in vivo. Therefore, these results underscore the protective role of *nmMLCK* deletion against an increased ileal permeability provoked by NASH fEVs. Importantly, fEVs from NASH patients evoked an early increase in intestinal permeability after one‐month of treatment in wild type mice and induced a trend increase in steatosis. Thus, we propose that they behave as a triggering effect at the early onset of the disease.

Next, we addressed the hypothesis that fEVs induce endothelial barrier dysfunction and inflammation. NASH fEVs that carried greater LPS quantities compared to NAFLD fEVs were able to increase the endothelial permeability and transmigration of monocytes in primary aortic endothelial cells. Pharmacological blockade of TLR4 activity prevented NASH fEV‐induced endothelial permeability and the transmigration of monocytes suggesting that fEVs activate the TLR4 pathway. In line with the present study, we reported recently that LPS‐enriched cEVs from metabolic syndrome patients contribute to the low‐grade inflammation in these patients and trigger endothelial dysfunction by activation of TLR4. Here, the increased endothelial permeability was associated with inflammation and production of cytokines, such as IL‐6, IL‐8, CCL2, G‐CSF, and GM‐CSF, in aortic endothelial cells. Conversely, pharmacological blockade of TLR4 activity tended to decrease the effect of NASH fEVs on IL‐6, IL‐8, CCL2 and CXCL1 production in endothelial cells. Together, our results suggest that NASH fEVs carried a greater LPS quantity and activated TLR4 to increase endothelial permeability, monocyte transmigration, and promote endothelial production of proinflammatory cytokines and chemokines, all of which concur with NASH development. The pathophysiological relevance of our study was evaluated on HHSECs, as they are the first liver cells to encounter fEVs. Indeed, G‐CSF expression was increased following NASH fEV stimulation suggesting that they also induce HHSEC inflammation.

Once fEVs attain the liver, they can be in contact with different cells. Interestingly, NASH fEVs altered hepatocyte integrity, as shown by the decrease in number of bile canaliculi using CMFDA fluorescence analysis. This effect was associated with a decrease in occludin and ZO‐1 protein levels. Thus, fEVs altered the integrity of hepatocytes tight junction. Moreover, fEVs can modulate the activation of stellate cells that is a key step in fibrogenesis (Tsuchida & Friedman, 2017). Notably, NASH fEVs, but not nNnN or NAFLD fEVs, were able to induce the expression of pro‐fibrotic proteins, collagen‐Iα and α‐SMA, and TGF‐β. In addition, NASH fEVs induced a greater production of several cytokines, notably CCL2, which specializes in macrophage recruitment in the liver. CCL2 has also been demonstrated to promote stellate cell activation and immune cell survival in the liver (Seki & Schwabe, 2015). The pathophysiological relevance is also provided because cEVs from NASH patients activate the expression of pro‐inflammatory cytokines and chemokines, including IL‐6, CCL2, MIF, and CXCL1 in stellate cells. NASH cEV stimulation did not significantly modify IL‐6, IL‐8, MCP1, MIF and/or Serpin1 production in Kupffer cells. These data reinforce the hypothesis that the presence of the microenvironment may be needed to observe the crosstalk between different cell types in the liver. Nevertheless, these results underscore that both, fEVs and cEVs from NASH patients participate in inflammation, liver injury, and, ultimately, fibrosis, at least partially, via altered hepatocyte integrity and stellate cell activation.

Finally, metabarcoding analyses were conducted in order to detect the potential differences in EVs produced by the gut microbial community between NAFLD and NASH patients. We did not observe any difference in terms of α‐diversity or β‐diversity between NAFLD and NASH fEV samples. At the phylum level, we observed that NAFLD and NASH fEV samples were mainly composed of gram‐positive Firmicutes and gram‐negative Bacteroidetes and Proteobacteria. While the latter phylum appeared overabundant in NASH fEVs, no statistically significant differences were observed between the two sample types. fEV bacterial origins are congruent with the gut microbiota composition described in NAFLD and NASH patients (Aron‐Wisnewsky et al., 2020; Loomba et al., 2017). Indeed, numerous studies have reported an increased abundance of Proteobacteria in NASH patients compared to healthy controls (Van Best et al., 2015). Furthermore, the apparent overabundance of the Proteobacteria phylum in NASH fEVs may be linked to a greater LPS quantity in these samples. We observed overabundant of 9 ASVs families or genera in NASH fEVs. An increased abundance of *Bacteroides* and *Ruminoccocus 1* genera has already been described in the gut microbial community of NASH patients (Boursier et al., 2016; Brandl & Schnabl, 2017). However, our results are contradictory to previous reports concerning *the Prevotella* genus and Ruminococcaceae family, both of which have been reported to be less represented in NASH patients (Boursier et al., 2016; Brandl & Schnabl, 2017; Henao‐Mejia et al., 2012). This finding is interesting, as it may suggest that, despite their lower abundance in the NASH gut microbiota community, they may play a part in NASH features through EV release. Moreover, *Prevotella*, gram‐negative bacteria, have been reported to exacerbate NASH progression in a methionine‐choline deficient mouse model, via TLR4/TLR9 pathways in the liver (Henao‐Mejia et al., 2012). Thus, we propose that the effects described in the present study may be mediated by *Prevotella* EVs transported from the intestine to the liver. Other ASVs have not been described in the gut microbiota composition of NASH patients so far. Focusing on NAFLD fEV samples, four ASVs were considered overabundant and belonged to *Ruminococcus 1*, *Romboutsia, Paraprevotella*, and Prevotellaceae family. The latter has been emphasized to be overabundant in patients suffering from minor liver injuries, compared to fibrotic patients (Aron‐Wisnewsky et al., 2020). Concerning *Romboutsia* and *Paraprevotella* genera, both have been reported to be associated with hepatic lipogenesis and fat accumulation in rats and obese adolescents, respectively (Stanislawski et al., 2018; Zhao et al., 2018). One can hypothesize that fEVs produced by these bacterial genera may be implicated in the development of steatosis during NAFLD.

**FIGURE 8:**
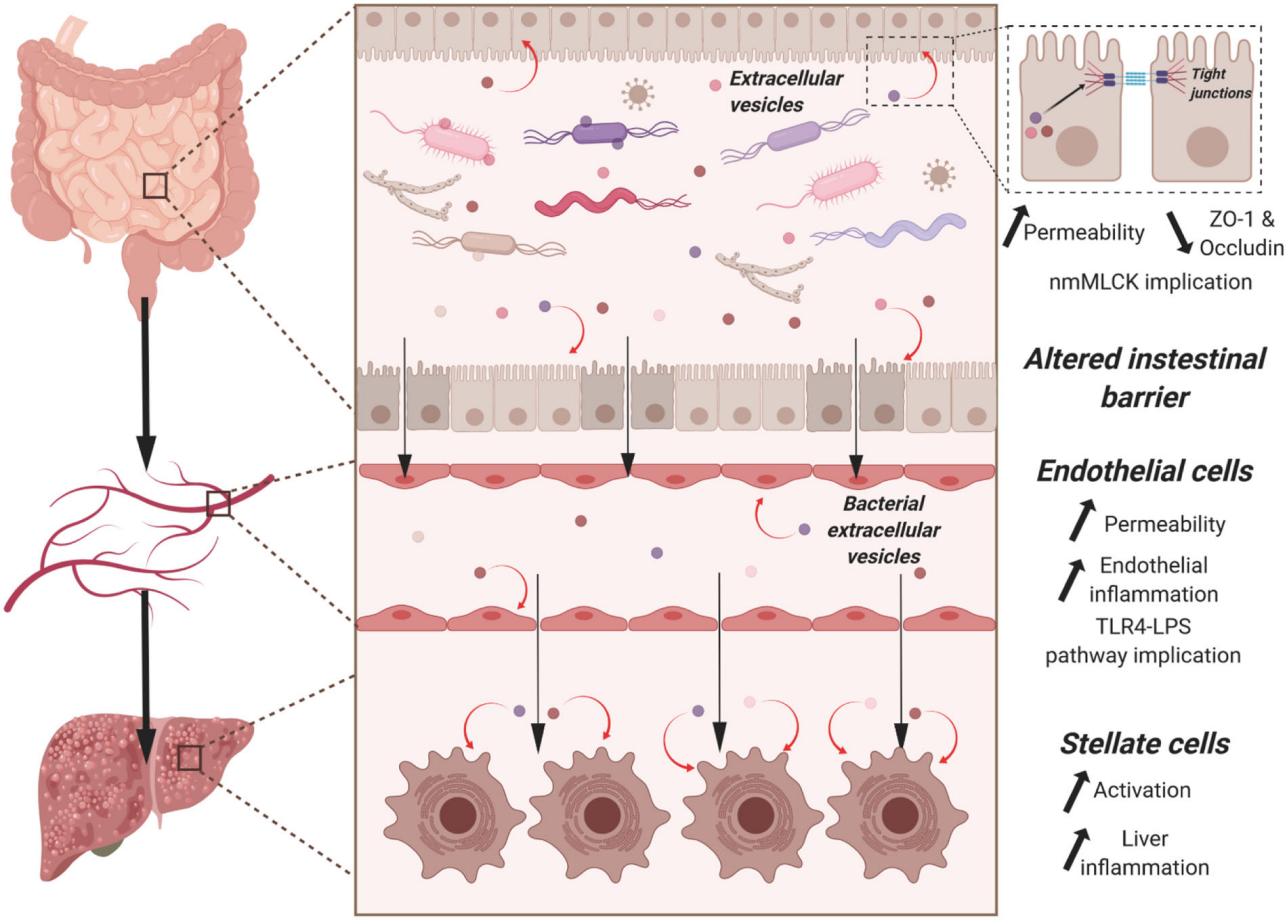
Graphical abstract summarizing the results and conclusion of the effects of NASH fEVs

In summary, we demonstrated that fEVs are a key player in barrier dysfunction, inflammation, and liver injuries especially in patients with NASH, at the early onset of the disease. fEV composition reflects the presence of bacterial origins that are congruent with the gut microbiota composition described in NAFLD and NASH patients, in addition to the presence of eukaryotic cells. fEVs from NASH patients are deleterious in inducing intestinal barrier dysfunction by targeting nmMLCK. fEVs cross the intestinal barrier to promote systemic inflammation via TLR4 action through LPS, and hepatic endothelial inflammation. Finally, fEVs participate in inflammation, liver injury and, ultimately, fibrosis, via stellate cell activation. Figure 8 summarizes the regulation mechanism and the conclusion of the present manuscript.

Limitations of the studies: it will be important to demonstrate the steatohepatitis exacerbating effects of fEVs in a mouse model of liver diseases at its early stage, which can be possibly blocked in nmMLCK‐ or TLR4‐deficient mice.

Together, our translational results support the potential role for fEVs as key players in NAFLD progression towards NASH, by acting from the gut to the liver, and underscore nmMLCK and TLR4 as potential key targets for NASH resolution and fibrosis.

## AUTHOR CONTRIBUTIONS

Michel Neunlist, Jérôme Boursier and Ramaroson Andriantsitohaina acquired the funding. Nadia Benabbou designed faeces‐extracellular vesicles purification method and performed preliminary in vitro experiments. Lionel Fizanne, Alexandre Villard, Nadia Benabbou, Raffaella Soleti, Sylvain Recoquillon, Xavier Vidal‐Gómez, M. Carmen Martínez and Ramaroson Andriantsitohaina designed the experiments and the study. Lionel Fizanne, Alexandre Villard, Sylvain Recoquillon, Raffaella Soleti, Mireille Wertheimer performed in vitro experiments. Lionel Fizanne, Alexandre Villard, Sylvain Recoquillon, Raffaella Soleti, Thibauld Oullier perform in vivo experiments. Lionel Fizanne, Alexandre Villard, Sylvain Recoquillon, Raffaella Soleti acquired and analysed in vitro and in vivo data. Alexandre Villard, Erwan Delage, Samuel Chaffron analysed and discussed metagenomics data. Lionel Fizanne, Alexandre Villard, Erwan Delage, Samuel Chaffron, M. Carmen Martínez and Ramaroson Andriantsitohaina wrote, reviewed and edited the manuscript.

## CONFLICT OF INTEREST

The authors declare no conflict of interests.

## Supporting information

Supplementary information
