# Supplementary material for: Faeces‐derived extracellular vesicles participate in the onset of barrier dysfunction leading to liver diseases
Source: J Extracell Vesicles. 2023 Jan 28;12(2):12303. doi: 10.1002/jev2.12303 (PMC9883837; doi:10.1002/jev2.12303)
Supplement: Supplementary file 1 — Supplementary information [file JEV2-12-12303-s001.docx]

**Feces-derived extracellular vesicles participate in the onset of barrier dysfunction leading to liver diseases**

Short title: “Extracellular vesicles and liver diseases”

Lionel Fizanne^1**^, Alexandre Villard^1,2**^, Nadia Benabbou^2†^, Sylvain Recoquillon^2†^ Raffaella Soleti^2†^, Erwan Delage^3^, Mireille Wertheimer^2^, Xavier Vidal-Gomez^2,4^, Thibauld Oullier^5^, Samuel Chaffron^3^, M Carmen Martinez^2,4^, Michel Neunlist^5^, Jérôme Boursier^1,6^ and Ramaroson Andriantsitohaina^2,4*^

Table of contents:

- Supplemental Methods …………………………………………………. 2
- Supplemental table 1.…………………………………………………...14
- Supplemental table 2.…………………………………………………...15
- Supplemental figure 1…………………………………………………...16
- Supplemental figure 2…………………………………………………...17
- Supplemental figure 3…………………………………………………...18
- Supplemental figure 4…………………………………………………...19
- Supplemental figure 5…………………………………………………...20
- Supplemental figure 6…………………………………………………...21
- Supplemental figure 7…………………………………………………...22
- References....…………………………………………………...............23

**Methods**

**Patients**A total of 95 patients with biopsy-proven NAFLD were recruited to the study at the Angers University Hospital, between October 2016 and September 2019. The study protocol conformed to the ethical guidelines of the current Declaration of Helsinki and was approved by the local ethics committee (SNIFF cohort initially approved by CPP OUEST II: 01/19/2010 (CB 2010-01)) and validated by the National Commission for Computing and Liberties 1998-001 on 12/20/2017. A total of 6 healthy individuals served as controls and were named as “non-NAFLD/non-NASH” (nNnN). Patients who had cirrhosis complications, history of chronic inflammatory bowel disease or bariatric surgery, excessive alcohol consumption (> 140 g/week for women and > 210 g/week for men) or received antibiotic treatment within 2 months before sampling were excluded from this study. All patients provided written informed consent before participating in the study.

**Histological criteria**

Liver biopsy was evaluated for steatosis, ballooning, lobular inflammation and fibrosis according to the NASH Clinical Research Network scoring system.^1^ NASH was defined as the presence of all three of the following conditions: steatosis grade ≥1, lobular inflammation grade ≥1, and ballooning grade ≥1. Fibrosis was staged as follows: F0 = no fibrosis, F1 = perisinusoidal or portal/periportal fibrosis, F2 = perisinusoidal and portal/periportal fibrosis, F3 = bridging fibrosis and F4 = cirrhosis. “Significant fibrosis” was defined as fibrosis stage F≥2, and “no/mild fibrosis” as F0-1.
***Isolation and characterization of feces-derived EVs (fEVs) and circulating small EVs***

*Isolation of fEVs*

Feces samples were collected and frozen at -80°C. Upon isolation, samples were thawed and aliquoted in several 1-gram samples. For each gram, 10 mL of sterile PBS was added and vortexed until the aggregates were dissolved. Four rounds of centrifugation were performed at 4°C: First, at 700 *g* for 15 minutes. Then, the supernatant was collected and centrifuged at 3,600 *g* for 15 minutes. Then, the supernatant was collected and centrifuged at 7,800 *g* for 15 minutes. Then, the supernatant was collected and centrifuged at 10,000 *g* for 30 minutes. The remaining supernatant was filtered using 0.45-µm- and 0.22-µm-filters and ultracentrifuged at 150,000 *g* for 2 hours at 4°C. The resulting pellet was washed with sterile PBS and ultracentrifuged using the same conditions once again. Each fEV pellet was resuspended in 200 µL of sterile PBS and stored at -80°C. The mean protein quantity of each fEV sample was assessed using a DC Protein Assay (Bio-Rad, Marnes-la-Coquette, France).

*Circulating small extracellular vesicle (cEV) isolation*

Blood samples were collected in EDTA K2 tubes and centrifuged at 260 *g* for 15 minutes. The plasma was collected and centrifuged at 1,500 *g* for 20 minutes. The resulting platelet-poor plasma (PPP) was collected and centrifuged at 21,000 *g* for 45 minutes. Supernatant was collected and ultracentrifuged at 100,000 *g* for 70 minutes at 4°C. Then, supernatant was discarded, and 200 µL of sterile PBS was added to the cEV pellet. The latter was homogenized before being ultracentrifuged at 100,000 *g* for 70 minutes at 4°C. The supernatant was discarded, and the cEV pellet was suspended in 50 µL of sterile PBS. The mean protein quantity of each cEVs sample was assessed using a DC Protein Assay (Bio-Rad).

***Transmission Electron Microscopy***

fEV samples were fixed in 2.5% glutaraldehyde overnight under gentle agitation. fEVs were washed in sterile PBS and ultracentrifuged during 2 hours at 150000 *g*. The pellet was re-suspended in sterile water before imaging. One µL of fixed-fEVs were placed on 150-mesh copper grids (Electron Microscopy Sciences, Hatfield, USA) and stained with phosphotungstic acid (1%, Merck, Darmstadt, Germany) during 1 minute before being washed extensively. Excess of liquid was removed on filter paper.

cEV samples were fixed in 1% of glutaraldehyde during 5 minutes before being placed on 150-mesh copper grids (Electron Microscopy Sciences). Grids were washed extensively before being stained with 0.4% of uranyl acetate (Merck, Darmstadt, Germany) and 2% of methylcellulose (Electron Microscopy Sciences) during 10 minutes on ice. Excess of liquid was removed on filter paper. Images were taken with a JEM 1400 microscope (JEOL, Akishima, Japan) at a voltage of 120KV.

***Nanoparticle Tracking Analysis***

fEVs and cEVs size and concentration were determined with a NanoSight NS300 (Malvern Instruments, Orsay, France) as described elsewhere.^2^ fEV and cEV samples were diluted to 1:500 or 1:100, respectively, in sterile NaCl 0.9% before analysis.

***LPS quantification in fEVs, PPP and cEVs***

Quantification of LPS in samples was performed by Pierce™ Chromogenic Endotoxin Quant Kit (Thermofisher scientific, Asheville, USA) following manufacturer’s instructions. fEV and cEV samples were diluted 50 or 20 times, respectively, in endotoxin-free water before measurement.

**Cellular studies**

All stimulations by fEVs were performed for 24 hours at a concentration of 1 µg protein/mL. This concentration represents the maximal active concentration to induce oxidative stress in intestinal epithelial cells, Caco-2 cells (data not shown). All stimulations by cEVs were performed for 24 hours at the circulating concentrations determined by nanoparticle tracking analysis of each corresponding patient. All vehicle stimulations were performed using sterile PBS.

*Primary cells and cell lines*

Caco-2 cells were purchased from ATCC (Manassas, USA). Human aortic endothelial cells (HAoECs) were purchased from Promocell (Heidelberg, Germany). Human hepatic sinusoidal endothelial cells (HHSECs) were purchased from Sciencell (Carlsbad, USA). The LX-2 cell line was licensed from Merck (Darmstadt, Germany). Kupffer cells were purchased from ABM (Paris, France).

***In vitro* permeability and integrity measurement**

To measure the epithelial permeability, Caco-2 cells were cultured in transwell (0.4 μm pore size, Corning, Corning, USA) as described elsewhere.^3^ Briefly, 5x10^5^ cells were maintained in transwells for 14 days post-confluence. Trans-epithelial electric resistance (TEER) measurement was performed using a Millicell® ERS-2 Voltohmmeter (Merck, Darmstadt, Germany) to monitor barrier integrity.^4^ To measure the endothelial permeability, HAoECs were cultured in transwells (0.4 μm pore size, Corning) until confluence. 2.5x10^5^ cells were seeded in the upper chamber. Measures were performed at three different points in each transwell and the mean was calculated. After stimulation of 24 hours by fEVs, TEER measures were performed once again, and results were normalized with measures obtained the day before stimulation. To assess toll-like receptor 4 (TLR4) implication on fEV-induced intestinal permeability, TLR4 inhibitor, TAK-242 (3 µM or 1 µM for Caco-2 and HAoECs, respectively) (Sigma-Aldrich, Saint-Louis, MO) was added to the apical compartment 30 minutes prior fEV stimulation. To assess the implication of non-muscular myosin light chain kinase (nmMLCK) in fEVs mediated effects, nmMLCK pharmacological inhibitor ML-7 (10 µM) (Sigma-Aldrich) was used. ML-7 was added to the apical compartment 30 minutes prior fEV stimulation. To measure the integrity in HepG2 cell model, 5-Chloromethylfluorescein diacetate (CMFDA) (Santa-Cruz) was used.^5^ Briefly, 8x10^4^ cells/well were plated in a µ-Slide 8 Well (Ibidi, Gräfelfing, Germany) and growth assessed for 4 days before fEV stimulation. After the stimulation with fEV, cells were washed with Hank's Balanced Salt Solution (HBSS), before an incubation for 30 minutes with CMFDA in HBSS (5µM). Afterwards, cells were washed with HBSS 3 times to restrain non-specific fluorescence, and fluorescence of bile canaliculi was assessed using confocal microscopy, with an LSM 700 microscope (Zeiss, Marly Le Roi, France). For each condition, three representative pictures were taken, and the number of bile canaliculi were counted using FIJI software.^6^

**Western Blot**

**For characterization of EVs**: Ten µg of EV-associated proteins were separated on 4-15% precast SDS-PAGE (Bio-Rad) and transferred on nitrocellulose membrane according to manufacturer indications and blocked during 1 hour in 5% BSA diluted in Tris-Buffered Saline 0.1% Tween. Primary antibodies anti-GPA33 (1:500, Thermofisher scientific, Waltham, MA), anti-CD81 (1:500, Santa-Cruz, Dallas, TX), anti-CD63 (1:500, Santa-Cruz); anti-Lipoteichoic Acid (LTA) (1:500, Novus Biologicals, Centennial, CO) and Anti-TSG 101 (1:500, Santa-Cruz) were used. Secondary antibodies coupled to peroxidase anti-rabbit (US Biological, Salem, MA), anti-mouse (Sigma-Aldrich) or anti-goat (Sigma-Aldrich) were used to detect bound proteins.

**For occludin expression in Caco-2**: Five x 10^5^ cells were plated in 12 wells plates and were maintained 14 days post-confluence before fEV stimulation for 24 hours. Cells were homogenized and lysed with RIPA lysis buffer. Twenty µg of proteins were separated on 4-15% precast SDS-PAGE and blocked during 1 hour in 5% BSA diluted in TBS-0.1% Tween. Primary antibody anti-occludin (1:50000, Abcam, Cambridge, UK) was used. For detection of total loading proteins, primary antibody anti-β-actin (1:5000, Sigma-Aldrich) or anti-glyceraldehyde-3-phosphate dehydrogenase (GAPDH) (1:3000, Santa-Cruz) was used. To assess TLR4-implication on occludin protein expression, TLR4 inhibitor, TAK-242 (3 µM) (Sigma-Aldrich) was added to the cells 30 minutes prior fEV stimulations.

**For protein expression in LX2**: Cells were harvested and lysed in RIPA lysis buffer. Twenty µg of proteins were loaded and separated on precast SDS-PAGE. Primary antibodies anti-α-1 type 1 collagen (1:1000, Cell Signaling, Danvers, MA), anti-transforming growth factor-β (TGF-β) (1:1000, Cell Signaling) and anti-α smooth muscle actin antibody (α-SMA) (1:500, Abcam) were used. For detection of total loading proteins, primary antibody anti-GAPDH was used.

**Immunofluorescence**Five x 10^5^ cells were seeded in 12-wells transwells (0.4 µm pore size, Corning, Corning, USA) and were maintained 14 days post-confluence before fEV stimulation for 24 hours. Then, transwells were fixed with 4% paraformaldehyde during 10 minutes at room temperature and blocked with 5% BSA for 30 minutes. ZO-1 antibody (1:3000, Abcam) was incubated overnight. Secondary antibody Alexa Fluor 488 goat anti-rabbit (1:3000, Abcam) was incubated for 1 hour. Transwells were cut out, mounted on glass with Dapi (ThermoFisher) staining and observed by confocal microscopy (LSM 700; Zeiss, Oberkochen, Germany). Images were acquired with ZEN software (Zeiss). The mean intensity of fluorescence was calculated with 3 representative pictures with FIJI software.^6^

**Transmigration assay**

To measure the endothelial permeability, HAoECs were cultured in transwells (8 μm pore size, Corning) until confluence. Once cells have reached confluence, fEV stimulation was performed for 24 hours. Then, the transmigration of monocytes through endothelial barrier was assessed. The monocytic cell line THP-1 (ATCC) was cultured in RPMI 1640 medium (Sigma-Aldrich) supplemented with 10% FBS and 1% antibiotics. THP-1 were stained by PKH-67 (Sigma-Aldrich) according to manufacturer instructions. Three x 10^5^ stained THP-1, in RPMI medium without FBS, were added to the apical compartment of the transwell and were allowed to transmigrate, to the basal compartment, for 24 hours. Basal medium was harvested, centrifuged 10 minutes at 500 *g* to pellet the cells and re-suspended in 200 µL of PBS. PKH-67-stained monocytes were counted with a flow cytometer FC 500 MPL (Beckman Coulter, Villepinte, France).

**Cytokine array**

Following fEV or cEV stimulation, cytokines/chemokines levels in culture medium of HAoECs, HHSECs, LX-2 and Kupffer cells were assessed using Proteome Profiler Human Cytokine Array Kit (R&D systems, Minneapolis, MN) following manufacturer indications. Signal intensity was measured by FIJI Software and “Protein Array Analyzer Plugin” as previously described.^7^ Each experiment was expressed as a percentage of variation compared to vehicle condition. Only proteins expression significantly different between fEVs samples were represented.

**Magnetic Luminex Assay**

Following fEV stimulation, IL-6, CCL2 and IL-1β levels in culture medium of HAoECs and LX-2 were measured using Human Premixed Multi-Analyte Kit Magnetic Luminex Assay (R&D systems) following manufacturer indications.

**Bacterial 16S rDNA extraction, amplification and sequencing**DNA was extracted from 100 µL of each fEVs sample using DNeasy PowerSoil Kit (Qiagen, Hilden, Germany). Bacterial DNA was amplified with 5’-CCTACGGGNGGCWGCAG-3’ and 5’-GACTACHVGGGTATCTAATCC-3’ primers, targeting hypervariable regions V3-V4 of 16S rDNA gene respectively. Sequencing was performed on a MiSeq System (Illumina, San Diego, USA) according to manufacturer’s instructions.

**Diversity and composition analysis**

MicroSysMics (https://bio.tools/microSysMics), a workflow built around the QIIME 2 toolbox,^8^ was used to analyze the microbiome data. PCR primers were removed with Cutadapt.^9^ Reads were then processed with Dada2.^10^ Both forward and reverse reads were truncated at length 220, to remove low quality ending bases while keeping a sufficient overlap for merging forward and reverse reads. Default parameters were used to discard reads with any ambiguous bases, having a number of expected errors greater than 2, or being shorter than 220 bases. Cleaned reads were denoised to correct for sequencing errors, infer ASV and estimate their abundance. Forward and reverse reads were merged and further curated by removing chimeras. Taxonomic classification was performed with the SILVA 132 99% 16S rRNA Naïve Bayes pre-trained classifier provided with QIIME2 (https://docs.qiime2.org/2019.10/data-resources/). Alpha and beta diversity metrics were calculated on a rarefied ASV table with a rarefaction sampling depth of 48880. Kruskal-Wallis and PERMANOVA tests were used to detect significant differences among groups for alpha and beta diversity, respectively.
Subsequent analyses, such as ASV agglomeration at higher taxonomic ranks, were performed using R packages phyloseq (1.32.0) and qiime2R (0.99.34).

**Differential abundance analysis**

Differential abundance analysis was conducted with DESeq2 (1.28.1) at genus, species and ASV levels. DESeq2 was originally developed to compare gene expression between different conditions on RNA-Seq data.^11^ However, it has also largely been applied to microbiome studies. Especially, it has been shown that DESeq2 has a better sensitivity over alternative methods when the number of samples per group is low.^12^ Abundance matrices were filtered to only retain features found in at least 3 samples of one of the conditions being tested. Normalization was done in “poscounts” mode which accounts for the inherent sparsity of microbiome data. Benjamini-Hochberg procedure with an FDR threshold of 0.05 was applied to account for multiple testing.

**16S rDNA detection in circulating EVs**

cEVs isolated from blood of non-NAFLD/non-NASH donors or NAFLD/NASH patients were filtered with a 0.22 µm filter and ultracentrifuged at 110,000 *g* for 70 minutes. cEV pellets were suspended in 100 µL of sterile 0.9% NaCl. Presence of 16S rDNA in samples was determined by PCR using primer for 16S variable regions V1-V3: 27f 5’-AGAGTTTGATCCTGGCTCAG-3’ and 534r 5’-ATTACCGCGGCTGCTGG-3’.^13^ Amplification conditions were 1 cycle of 94°C for 3 minutes, 34 cycles of 94°C for 30 seconds; 56°C for 1 minutes; 72°C for 1’15 minutes, and 1 cycle of 72°C for 10 min using a Bio-Rad CFX384 Real-time system. Migration of amplicons was performed using a 2% agarose gel.

**Animal experiments**

All procedures were carried out in accordance with the guidelines and authorization by the French Ministry of Agriculture regulations based on the European Community and were approved by the local ethics committee “Comité d’éthique en expérimentation animale Pays de la Loire”; Apafis#320027-2019032910558370v5. Eight-week-old wild-type C57BL6 male mice (Janvier Labs, Le Genest-Saint-Isle, France) or non-muscular myosin light chain kinase (*nmMLCK*)*^-/-^* mice generated as previously described,^14^ were maintained at a room temperature of 23°C under a 12-h light/dark cycle and were allowed free access to food and water throughout the experiment. Mice were intragastrically administered 1 µg of fEV proteins diluted in sterile PBS 3 times a week for one month, under blinding conditions. Following euthanasia, the liver and intestine were collected for further analysis.

***In vivo* intestinal permeability measurement**

Following fEV treatment, the mice were euthanized, and their intestines were collected. Intestinal permeability was measured using an Ussing chamber, as previously described.^15^ Briefly, the jejunum, ileum, proximal colon, and distal colon were separated, and segments of each tissue were mounted on a Ussing chamber. Fluorescein–5.6 sulfonic acid (1 mg/mL, Invitrogen, Illkirch, France) was added to the apical medium, and the fluorescence intensity in the basal medium was measured every 30 min for 180 min. Fluorescence intensity was measured using a Varioskan fluorimeter (Thermofisher). Fluorescein–5.6 sulfonic acid flow was measured using a linear regression ﬁt model for each tissue.

**Histology and analysis of steatosis and fibrosis**

A histological assessment of the livers has been performed on formalin- fixed paraffin-embedded tissue sections after staining with hematoxylin-eosin (H&E) and red picrosirius staining. Histological grading of fibrosis was based on the Metavir grading system: The fibrosis staging of the Metavir staging was as follows: F0: no fibrosis, F1: enlarged portal tract without septa, F2: enlarged portal tract with rare septa, F3: numerous septa without cirrhosis, F4: cirrhosis.

*Quantification of collagen content*

Collagen content quantification was performed on formalin fixed paraffin-embedded tissue sections after staining with red picrosirius Red-positive areas were measured using image analysis.

*Image acquisition*

For each picrosirius red-stained section of the whole liver biopsy was scanned using the Aperio Scanscope® CS System (Aperio Technologies, Vista, CA, USA). The image processor provided high-quality images of 30 000 × 30 000 pixels and a resolution of 0.5 μm/pixel. The operator manually suppressed artifacts (such as folds or dust) or liver capsule (if present) on digital images and measured the digital liver specimen’s length. A binary image (black and white) of the whole specimen was obtained by automatic thresholding of the red pixels using an algorithm developed in our laboratory.

*Areas of Fibrosis and Steatosis*

The area of whole fibrosis was measured on the binary image with the use of ImageJ software V.1.41o. (imagej.nih.gov/ij/). We developed an automated segmentation algorithm to separately detect and quantify the portal and/or bridging fibrosis (referred as the area of portal–bridging fibrosis), therefore bypassing the selection of areas of interest by a pathologist. The area of perisinusoidal fibrosis relative to the lobular area was calculated by the software from all the pixels in perisinusoidal fibrosis (named PixsF) and the entire lobular area (named PixLa,), according to the formula: area of perisinusoidal fibrosis=PixsF/PixLa × 100. Any fibrous septum lacking liver cell plates between collagen fibers was included in the area of portal–bridging fibrosis. The walls of the centrolobular veins whose caliber was <200 μm were automatically included in the area of perisinusoidal fibrosis in order not to miss small expansions of perisinusoidal fibrosis abutting veinular walls, whereas larger centrolobular veins were not included in that area. The automated quantification was validated by successive steps that compared, qualitatively and quantitatively, an interactive portal and bridging fibrosis selection with the automated segmentation. AOS (%) was calculated as the ratio: area of steatosis vesicles / complete liver surface, and relative AOS (rAOS, %) as the ratio: area of steatosis / non-fibrous liver area (i.e. complete liver surface minus AOF). Finally, the box counting method was used to evaluate the Kolmogorov FD of steatosis.

**Statistical analysis**

Data are represented as mean ± SEM. Values of P < 0.05 were considered statistically significant. P-values were adjusted using the Benjamini-Hochberg procedure. Each point of figures represents the number of animals. Data were compared across groups using the Kruskal-Wallis test followed by Dunn’s test for multiple comparisons. All analyses were performed using Prism software (V.8.0.2, GraphPad Software, La Jolla, CA).

|  | **nNnN** | **NF** | **NS** | **p. value** |
| --- | --- | --- | --- | --- |
| **Patients (n)** | 6 | 27 | 68 | - |
| **Age (Years)** | 38 (25-48) | 58 (28-78) | 58 (22-78) | 0.008^a^  0.002^b^  0.621 |
| **Sex (M/F)** | 3/3 | 23/4 | 48/20 | 0.148 |
| **Steatosis** |  |  |  |  |
| Grade 0 |  | 0 |  |  |
| Grade 1 |  | 18 | 28 | 0.028 |
| Grade 2 |  | 6 | 24 |  |
| Grade 3 |  | 3 | 16 |  |
| **Ballooning** |  |  |  |  |
| None (0) |  | 22 |  |  |
| Moderate (1) |  | 4 | 40 | <0.0001 |
| Severe (2) |  | 1 | 28 |  |
| **Lobular inflammation** |  |  |  |  |
| None (0) |  | 15 |  |  |
| Moderate (1) |  | 10 | 59 | <0.0001 |
| Severe (2) |  | 2 | 9 |  |
| **Fibrosis** |  |  |  |  |
| Stage 0 |  | 7 | 4 |  |
| Stage 1 |  | 10 | 9 |  |
| Stage 2 |  | 6 | 21 | <0.0001 |
| Stage 3 |  | 4 | 26 |  |
| Stage 4 |  | 0 | 8 |  |
| **Scores** |  |  |  |  |
| NAS (%) |  |  |  |  |
| NAS_0–2 |  | 18 |  | <0.0001 |
| NAS_3–4 |  | 9 | 41 |  |
| NAS_5–8 |  | 0 | 27 |  |
| **ALT (IU/l)** | 18.7 + 1.15 | 40 ± 5.6 | 65 ± 10 | 0.0006^b^ |
| **AST (IU/l)** | 22.5 + 3.2 | 29 ± 3.3 | 45 ± 5.4 | 0.01^b^ |
| **ɣ-GT (IU/l)** |  | 82 ± 17 | 125 ± 21 | 0.531 |
| **ALP (IU/l)** |  | 65 ± 6 | 76 ± 4.5 | 0.222 |

**Supplemental table 1:** Characteristics of human cohort. Analyses were performed using Kruskal-Wallis test followed by Dunn’s test for multiple comparisons or by Mann-Whitney test. nNnN: non-NAFLD/non-NASH patients matched in aged with NAFLD and NASH patients; NF: NAFLD; NS: NASH; ALT: Alanine transaminase; AST: Aspartate transaminase; Ɣ-GT: gamma-glutamyltransferase; ALT: alanine aminotransferase. a, NF *vs* nNnN; b, NS *vs* nNnN.

**Supplemental table 2:** Sixteen amplicon sequence variants differentially expressed between NAFLD and NASH feces-derived extracellular vesicles samples obtained using DESeq 2. lfcSE: standard error. stat: Wald statistic. p value: Wald test p-value. padj: Benjamini-Hochberg adjusted p-value.

**Supplemental figure 1:** (A) IL-6 and (B) Chemokine (C-C motif) ligand 2 (CCL2) were measured following feces-derived extracellular vesicles (fEVs) stimulation of human aortic endothelial cells (HAoECs) using the multiplex assay. (C) IL-6, (D) IL-8, (E) CCL2 and (F) CXCL1 were measured using cytokine array, in absence or presence of the nmMLCK inhibitor, ML-7 (10 µM), or the TLR4-pathway inhibitor, TAK-242 (3 µM). Statistical analyses were performed using Kruskal-Wallis test followed by Dunn’s test for multiple comparison. nNnN: non-NAFLD/non-NASH; NF: NAFLD; NS: NASH.

**Supplemental figure 2:** (A) HepG2 tight junction integrity assessed as the number of bile canaliculi retaining 5-Chloromethylfluorescein diacetate after stimulation with either vehicle (VL), feces-derived extracellular vesicles from NAFLD (fEVs NF) or NASH (fEVs NS) patients. Western blot and quantification of ZO-1 (B) and occludin (C) protein levels following fEV stimulation. Statistical analyses were performed using Kruskal-Wallis test followed by Dunn’s test for multiple comparison. Vehicle; NF: NAFLD; NS: NASH.

**
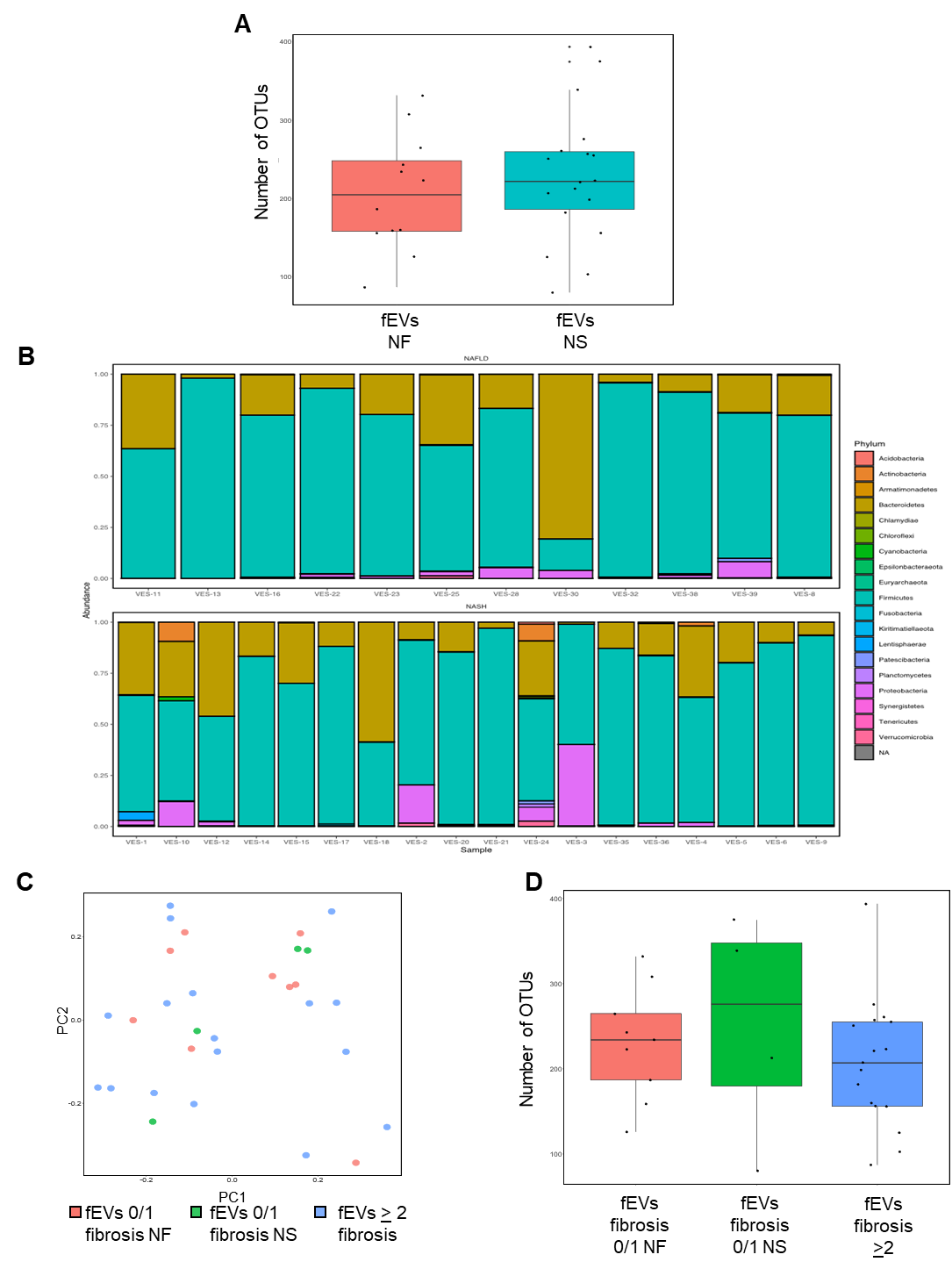
**

**Supplemental figure 3:** Metagenomic analyses of feces-derived extracellular vesicles (fEVs) samples. (A) Richness of fEVs from NAFLD (NF) and NASH (NS) patients (B) Relative abundance in each fEVs sample at the phylum level. (C) Principal coordinates analysis (PCoA) of dissimilarity measured by Bray-Curtis following fibrosis score. (D) Richness of fEVs from following fibrosis score. Amplicon sequence variants (ASVs) are here either identified by their genus or, when unassigned, their family. Statistical analyses were performed using Mann-Whitney test or Kruskal-Wallis test followed by Dunn’s test for multiple comparisons. OTUs: operational taxonomic units.

**
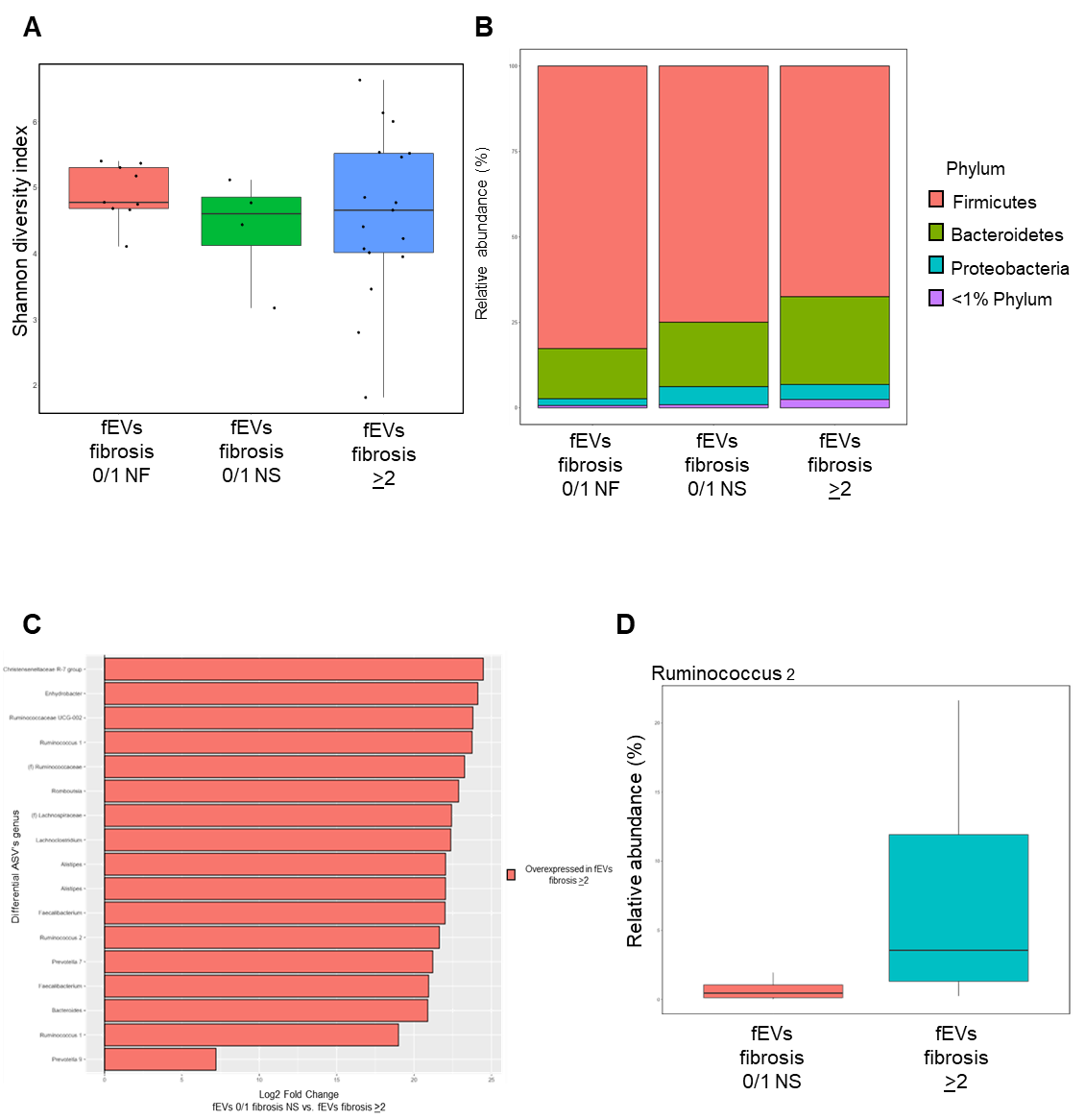
**

**Supplemental figure 4:** Metagenomic analyses of feces-derived extracellular vesicles (fEVs) samples indicates modifications in the fEVs-producing gut microbial community gathered by fibrosis score. (A) Alpha diversity between fEVs samples was measured by Shannon diversity index following fibrosis score. (B) Relative abundance in fEVs at the phylum level. (C) Differentially abundant amplicon sequence variants (ASVs) in NAFLD (NF) vs NASH (NS) samples. (D) Relative abundance of Ruminococcus 2 genus between fEVs samples of NS 0/1 fibrosis and fibrosis >2 patients. ASVs are here either identified by their genus or, when unassigned, their family. Statistical analyses were performed using Kruskal-Wallis test followed by Dunn’s test for multiple comparisons or Mann-Whitney test.

**
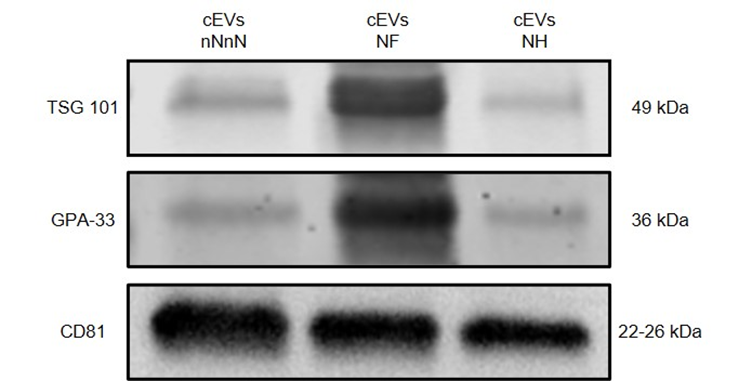
**

**Supplemental figure 5:** Characterization of circulating small extracellular vesicles (cEVs) from non-NAFLD/non-NASH (nNnN) donors and NAFLD (NF) /NASH (NS) patients. Representative western blot showing the presence of tumor susceptibility gene 101 (TSG 101), cell surface A33 antigen (GPA-33) and CD81 in cEV samples.

**Supplemental figure 6:** Cytokines and chemokines quantifications in supernatant from Kupffer cells. IL-6 (A), IL-8 (B), MCP-1 (C), MIF (D), CXCL1 (E), and Serpin-1 (F) quantification. (A-F) were measured following circulating-derived extracellular vesicles (cEVs) stimulation of Kupffer cells using the multiplex assay. (n = 3 to 4). Statistical analyses were performed using Mann-Whitney test. nNnN: non-NAFLD/non-NASH; NS: NASH.

**
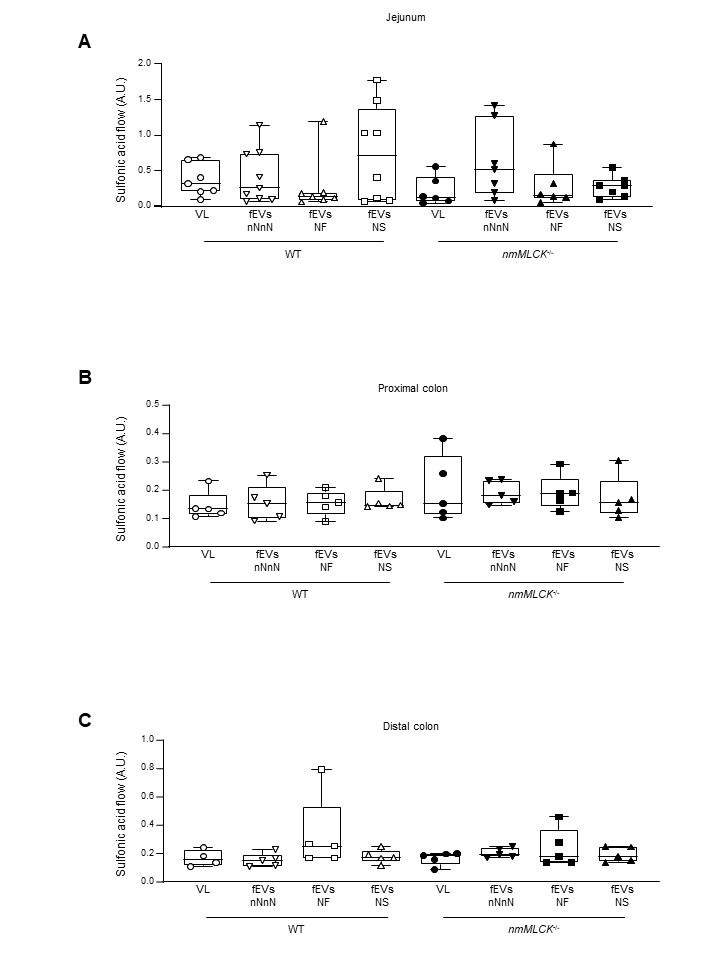
**

**Supplemental figure 7:** Measurements of jejunum, proximal colon and distal colon permeability *in vivo*. Wild-type (WT) or *nmMLCK*^-/-^ mice (n = 6 to 8) were orally force-fed three times a week with 1 µg of feces-derived extracellular vesicles (fEVs)-associated proteins three times a week for four weeks. Mice were then sacrificed, and their intestines were collected before measuring the intestinal permeability using an Ussing chamber. (A-C) The permeability of the jejunum (A), proximal colon (B) and distal colon (C) of WT and *nmMLCK*^-/-^ mice was assessed by measuring the sulfonic acid flow through the tissue section. VL: vehicle; nNnN: non-NAFLD/non-NASH; NF: NAFLD; NS: NASH.

**References:**

1. Kleiner DE, Brunt EM, Natta MV, *et al.* Design and validation of a histological scoring 632 system for nonalcoholic fatty liver disease. *Hepatology* 2005;**41**:1313-21. 633 doi:10.1002/hep.20701

2. Ali S, Malloci M, Safiedeen Z, *et al*. LPS-enriched small extracellular vesicles from metabolic syndrome patients trigger endothelial dysfunction by activation of TLR4. *Metabolism*. 2021;**118**:154727. doi: 10.1016/j.metabol.2021.154727.

3. Lea T. Caco-2 Cell Line. In: Verhoeckx K, Cotter P, López-Expósito I, *et al.*, eds. *The Impact of Food Bioactives on Health*. Cham: Springer International Publishing 2015. 103–11. doi:10.1007/978-3-319-16104-4_10

4. Srinivasan B, Kolli AR, Esch MB, *et al.* TEER measurement techniques for in vitro barrier model systems. *J Lab Autom* 2015;**20**:107-26. doi:10.1177/2211068214561025

5. Mee CJ, Harris HJ, Farquhar MJ, *et al*. Polarization restricts hepatitis C virus entry into HepG2 hepatoma cells. *J Virol* 2009;**83**:6211-21. doi:10.1128/JVI.00246-09

6. Schindelin J, Arganda-Carreras I, Frise E, *et al.* Fiji: an open-source platform for biological-image analysis. *Nat Methods* 2012;**9**:676-82. doi:10.1038/nmeth.2019

7. Rahimi F, Murakami K, Summers JL, *et al.* RNA aptamers generated against oligomeric Aβ40 recognize common amyloid aptatopes with low specificity but high sensitivity. *PLOS ONE* 2009;**4**:e7694. doi:10.1371/journal.pone.0007694

8. Bolyen E, Rideout JR, Dillon MR, *et al.* Reproducible, interactive, scalable and extensible microbiome data science using QIIME 2. *Nat Biotechnol* 2019;**37**:852-7. doi:10.1038/s41587-019-0209-9

9. Martin M. Cutadapt removes adapter sequences from high-throughput sequencing reads. *EMBnet.journal* 2011;**17**:10-2. doi:10.14806/ej.17.1.200

10. Callahan BJ, McMurdie PJ, Rosen MJ, *et al.* DADA2: High-resolution sample inference from Illumina amplicon data. *Nat Methods* 2016;**13**:581-3. doi:10.1038/nmeth.3869

11. Love MI, Huber W, Anders S. Moderated estimation of fold change and dispersion for RNA-seq data with DESeq2. *Genome Biol* 2014;**15**:550. doi:10.1186/s13059-014-0550-8

12. Weiss S, Xu ZZ, Peddada S, *et al.* Normalization and microbial differential abundance strategies depend upon data characteristics. *Microbiome* 2017;**5**:27. doi:10.1186/s40168-017-0237-y

13. Walker AW, Martin JC, Scott P, *et al.* 16S rRNA gene-based profiling of the human infant gut microbiota is strongly influenced by sample processing and PCR primer choice. *Microbiome* 2015;**3**:26. doi:10.1186/s40168-015-0087-4

14. Wainwright MS, Rossi J, Schavocky J, *et al*. Protein kinase involved in lung injury susceptibility: Evidence from enzyme isoform genetic knockout and in vivo inhibitor treatment. Proc Natl Acad Sci U S A. 2003;**100**:6233-8. doi:10.1073/pnas.1031595100

15. Tasselli M, Chaumette T, Paillusson S, *et al.* Effects of oral administration of rotenone on gastrointestinal functions in mice. *Neurogastroenterol Motil* 2013;**25**:e183-93. doi:10.1111/nmo.12070
